# Assembly and Repair of Photosystem II in *Chlamydomonas reinhardtii*

**DOI:** 10.3390/plants13060811

**Published:** 2024-03-12

**Authors:** Himanshu S. Mehra, Xiaozhuo Wang, Brandon P. Russell, Nidhi Kulkarni, Nicholas Ferrari, Brent Larson, David J. Vinyard

**Affiliations:** Department of Biological Sciences, Louisiana State University, Baton Rouge, LA 70803, USA; hmehra1@lsu.edu (H.S.M.); xwan128@lsu.edu (X.W.); bruss28@lsu.edu (B.P.R.); nkulka1@lsu.edu (N.K.); nferr11@lsu.edu (N.F.); blarso5@lsu.edu (B.L.)

**Keywords:** *Chlamydomonas reinhardtii*, photosystem II, assembly, repair

## Abstract

Oxygenic photosynthetic organisms use Photosystem II (PSII) to oxidize water and reduce plastoquinone. Here, we review the mechanisms by which PSII is assembled and turned over in the model green alga *Chlamydomonas reinhardtii*. This species has been used to make key discoveries in PSII research due to its metabolic flexibility and amenability to genetic approaches. PSII subunits originate from both nuclear and chloroplastic gene products in *Chlamydomonas*. Nuclear-encoded PSII subunits are transported into the chloroplast and chloroplast-encoded PSII subunits are translated by a coordinated mechanism. Active PSII dimers are built from discrete reaction center complexes in a process facilitated by assembly factors. The phosphorylation of core subunits affects supercomplex formation and localization within the thylakoid network. Proteolysis primarily targets the D1 subunit, which when replaced, allows PSII to be reactivated and completes a repair cycle. While PSII has been extensively studied using *Chlamydomonas* as a model species, important questions remain about its assembly and repair which are presented here.

## 1. Introduction

In oxygenic photosynthesis, visible light is used to energize electrons stripped from water. Simultaneously, protons are pumped across a membrane generating proton motive force. The final products, NADPH and ATP, are used for cellular functions including the fixation of CO_2_ in the Calvin–Benson Cycle (reviewed in [1]).

Photosystem II (PSII) is the first component of the photosynthetic electron transport chain and acts as a water–plastoquinone (PQ) oxidoreductase (see [2,3]). PSII is a large membrane-bound complex consisting of approximately 20 unique protein subunits [4]. Within the PSII core, the P_680_ primary chlorophyll-*a* donor undergoes charge separation upon photoexcitation. On the donor side of PSII, the hole in the ground state of P_680_^+^ is filled by a redox-active tyrosine, Y_Z_, which, is in turn, reduced by the Mn_4_CaO_5_ oxygen-evolving complex (OEC). Following four one-electron oxidation events, the OEC catalyzes the formation of O_2_ from two molecules of water. On the acceptor side of PSII, the excited electron in P_680_* is transferred to a pheophytin, a primary PQ acceptor, Q_A_, and then to a secondary PQ acceptor, Q_B_ (reviewed in [5,6]).

PSII serves as a model system for multiple scientific fields. Protein biochemists study PSII to learn how membrane protein complexes assemble and function (see [7,8]). Bioinorganic chemists have been intrigued by the multiple metal cofactors in PSII including the OEC (see [9]), cytochrome(s) (see [10]) and a non-heme iron (see [11]). Biophysical chemists and biophysicists use PSII to study visible-to-chemical energy conversion and exciton and electron transfer reactions (see [12]). Molecular biologists have used differentially expressed PSII subunits to learn about bacterial and plastidal gene regulation (see [13]) and have used PSII to study protein turnover mechanisms (see [14]). Evolutionary biologists analyze the changes (or lack of changes) in PSII subunits from cyanobacteria to plants (see [15]). Geologists recognize the role of PSII as the sole biological source of O_2_ from water oxidation (see [16]). PSII’s ability to efficiently perform this reaction using only light as an energy input inspires synthetic chemists, materials chemists, and engineers to attempt to replicate this activity (see [17]). This incomplete list illustrates the significant interest in PSII and its application to multiple research areas.

PSII and other photosynthetic complexes are generally conserved from cyanobacteria to algae to plants, and researchers in this field use all three groups as model species. For example, many biophysical studies of PSII have used membrane preparations from market spinach [18,19,20,21]. The cyanobacterium *Synechocystis* sp. PCC 6803 has been widely used to study mutations in PSII subunits [22,23,24,25,26,27]. This mesophilic species is naturally transformable and has highly efficient homologous recombination making it a very practical genetic system [28,29]. Detergent-solubilized PSII core complexes from thermophilic cyanobacteria such as *Thermosynechococcus vulcanus* have been used for multiple influential structural studies [30,31,32] and biophysical studies [33,34,35,36]. While all this work is valuable, the structures [37] and assembly and repair mechanisms [7] of PSII are not identical between oxygenic phototrophs.

Over the past seventy years, the model green alga *Chlamydomonas reinhardtii* (hereafter *Chlamydomonas*) has been used to elucidate many discoveries in photosynthesis and other fields (see [38,39]). As a unicellular eukaryote, *Chlamydomonas* is a practical model species for studying chloroplast biology. First, this species can grow photoautotrophically, mixotrophically, or heterotrophically. This trait allows *Chlamydomonas* to be studied with genetic mutations in photosynthesis genes that would otherwise be lethal. In addition, *Chlamydomonas* can reproduce asexually or sexually. In the research laboratory, cultures are typically maintained vegetatively. However, sexual reproduction facilitates genetic approaches. Because of these features, *Chlamydomonas* has been and is used to discover key components and mechanisms of PSII function, assembly, and repair.

Here, we review research studies that have used *Chlamydomonas* to gain insights into PSII. We aim to highlight the influential role of this organism in multiple scientific fields that all use PSII as a model system. We compile PSII-specific data to facilitate future studies and bring attention to areas where more research is needed.

## 2. Discussion

### 2.1. Architecture of the Chlamydomonas chloroplast

**Figure 1:**
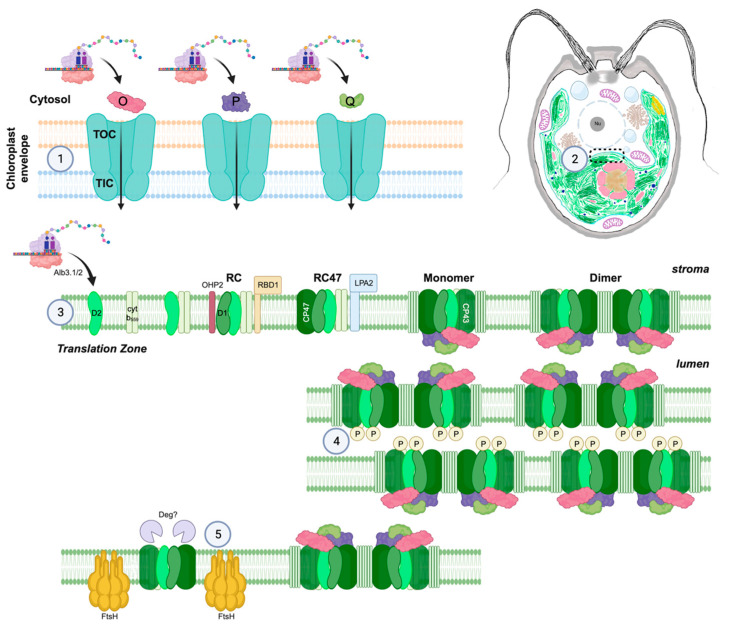
An overview of PSII assembly and organization in *Chlamydomonas*. ① Nuclear-encoded PSII subunits, including PsbO, PsbP, and PsbQ, are translated in the cytosol and imported into the chloroplast using the TOC TIC system. ② These complexes are spatially aligned with the chloroplast translation zone near the pyrenoid. ③ PSII subunits assemble in an organized pathway from reaction center intermediates to mature dimers. ④ Active and phosphorylated PSII dimers are enriched in appressed domains of the thylakoid membranes. ⑤ PSII assembly and degradation occur in non-appressed domains of the thylakoid membranes. Additional details and references are provided in the text.

In algae and plants, photosynthesis occurs in the chloroplast. *Chlamydomonas* cells develop one cup-shaped chloroplast occupying almost half of the volume of the cell [40,41] (Figure 1). The light-dependent photosynthetic reactions are localized to a network of membranes called thylakoids. These thylakoids are organized in appressed regions (regularly spaced stacks termed grana in land plants) and non-appressed regions. In *Chlamydomonas*, active dimeric PSII complexes are organized in appressed thylakoids, and Photosystem I and ATP synthase are localized to non-appressed regions [42,43].

The chloroplast emerged from the endosymbiosis of a cyanobacterium. Over the course of evolution, most of the genetic information from the original cyanobacterial genome was transferred to the host nuclear genome. In *Chlamydomonas*, only 72 unique protein-encoding genes are retained in the chloroplast genome [44]. As discussed below, most PSII subunits are chloroplast encoded. The synthesis of chloroplast-encoded photosystem proteins is concentrated at the Translation or T-zone, which is located near the pyrenoid [45,46,47] (Figure 1). Here, newly synthesized proteins are inserted directly into the membrane [48], which is facilitated by a complex containing Alb3.1 and Alb3.2 [49,50]. The *Chlamydomonas* chloroplast is a complex organelle with multiple sub-organellular structures [51].

**Figure 2:**
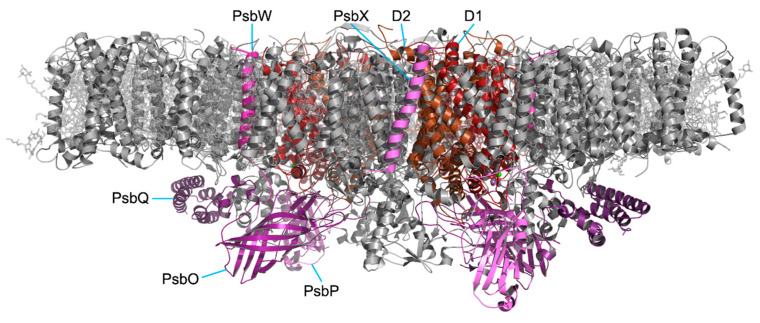
The PSII C_2_S_2_ complex from *Chlamydomonas* from (PDB ID 6KAC) [54]. Nuclear-encoded PSII subunits are shown in shades of purple.

Nuclear-encoded chloroplast proteins (including *Chlamydomonas* PSII subunits PsbO, PsbP, PsbQ [52], PsbW [53], and PsbX [54] (Figure 2)), are translated in the cytosol [55], targeted to the chloroplast [56], and then imported across the outer and inner chloroplast membranes using energy from ATP hydrolysis [57]. Chloroplast protein transport is facilitated by the translocon of the outer membrane (TOC) and translocon of the inner membrane (TIC) protein complexes [58,59,60,61] (Figure 1). Interestingly, the TOC and TIC complexes spatially align with the T-zone suggesting a highly coordinated system of protein import and translation in the *Chlamydomonas* chloroplast [62,63,64].

### 2.2. Transcription of PSII Subunits in the Chloroplast

The chloroplast genome encodes 16 of the 21 PSII subunits in *Chlamydomonas* [65] (see Figure 2). The most prevalent chloroplast transcript is *psbA* which encodes the PSII core subunit D1 [66,67]. However, little of this *psbA* mRNA is associated with ribosomes [68], indicating the tight control of translation initiation. The second most prevalent chloroplast transcript is *psbD* which encodes the D2 core subunit and is found at levels approximately five-fold less than *psbA* [65].

The PSII cytochrome b_559_ subunit is encoded by two genes, *psbE* and *psbF*. As described below, cytochrome b_559_ plays crucial roles in PSII translation control and assembly. In cyanobacteria [69], some algae [70,71], and plants [72], these genes are organized as part of a *psbEFLJ* operon. Surprisingly, in *Chlamydomonas*, *psbE* and *psbF* are separated and have reverse orientations [73,74]. *psbF* and *psbL* remain associated with each other and are co-transcribed [73]. *psbJ* is co-transcribed as part of a cluster that includes *psbD* [75,76].

In *Arabidopsis*, a mitochondrial transcription termination factor, mTERF5, controls the transcription of the *psbEFLJ* operon [77]. This regulatory mechanism is likely not conserved in *Chlamydomonas* given the differences in gene organization. Why *Chlamydomonas* evolved to have this unique cytochrome b_559_ expression system is not understood.

### 2.3. Translation of PSII Subunits in the Chloroplast—Control by Epistasy of Synthesis

The de novo synthesis of specific PSII subunits is regulated by the presence or absence of other PSII subunits through a mechanism termed Control by Epistasy of Synthesis (CES) (reviewed in [78]). Here, we discuss CES in *Chlamdyomonas* PSII assembly, but this mechanism is more general. In *Chlamydomonas*, CES has been observed in the assembly of cytochrome b_6_f, ATP synthase, ribulose-1,5-bisphosphate carboxylase/oxygenase (Rubisco), and PSI. It is also involved in the synthesis of Rubisco in plants and Cox1 in yeast mitochondria (see [79]).

In *Chlamydomonas*, the presence of the D2 subunit is required for D1 translation [80,81]. Subsequently, the presence of the D1 subunit is required for CP47 (*psbB*) translation [81]. This regulation is based on negative regulation of each unassembled polypeptide interacting with its own mRNA in the 5′ untranslated region (UTR) [68].

Regulation of cytochrome b_559_ translation in *Chlamydomonas* is more complicated. In a Δ*psbE* strain, the translation of D1, D2, CP43, and CP47 was not observed or was strongly inhibited [82]. As discussed below, cytochrome b_559_ is a component of early PSII assembly intermediates. The phenotypes observed in Δ*psbE* may reflect true CES and/or a defect in the assembly pathway.

### 2.4. Translation of PSII Subunits in the Chloroplast—Regulatory Elements

In the chloroplast, regulation occurs mostly at the translational level and requires both cis- and trans-regulating elements (see [83]). All characterized cis-regulatory elements are in the 5′ UTR of the genes [84,85,86]. Trans-regulating elements (translational activators) are crucial components of PSII core subunits’ translational control and are summarized in Table 1.

Clearly, the translational control of chloroplast-encoded PSII subunits is extensive. Table 1 is very likely incomplete, and more research is needed in this area. While trans-regulatory elements are discussed here for *Chlamydomonas*, similar regulation is present in plants (see [107]). In contrast to the chloroplast, cyanobacteria generally control PSII expression at the transcriptional level (see [108]).

### 2.5. Assembly of Protein Subunits and Cofactors

PSII subunits form discrete subcomplexes before assembling into monomeric then dimeric reaction centers (illustrated in Figure 1). First, D2 binds cytochrome b_559_ subunits PsbE and PsbF. D1 is then translated and binds with other subunits. The binding of the D1 subcomplex to D2-cytochrome b_559_ leads to the formation of the first reaction center (RC) complex. In *Chlamydomonas*, One-Helix Protein 2 (OHP2) stabilizes D1 during its translation by promoting chlorophyll association [109]. Another assembly factor, RBD1, promotes D1 stabilization [110,111] and is also involved in the delivery and/or reduction of the non-heme iron ion near the stromal surface of PSII [112]. Next, CP47 is translated and binds to create the RC47 complex. The binding of CP43, facilitated by the assembly factor LPA2 [113,114], low molecular weight subunits, and extrinsic subunits forms monomeric PSII.

After the PSII core is assembled, the OEC is assembled from Mn^2+^, Ca^2+^, and water in a stepwise process termed photo-assembly (reviewed in [115]). Here, metal ions and water molecules bind to the apo-OEC PSII protein. Light-driven oxidation events lead to higher valent Mn ions and the OEC cluster spontaneously assembles in situ. This process has mostly been studied in plants and cyanobacteria, but some groups have conducted experiments on *Chlamydomonas* whole cells [116] or isolated membranes [117]. The kinetics and efficiency of OEC photo-assembly are similar among these organisms suggesting a conserved mechanism [116,117].

The binding of the extrinsic subunits PsbO, PsbP, and PsbQ occurs late in the assembly process. (Note that the terms OEE1, OEE2, and OEE3, respectively, have also been used for these proteins [81,84,118]). PsbO is required for oxygen evolution and photoautotrophic growth in *Chlamydomonas* and acts by stabilizing the OEC [118]. This subunit is conserved in cyanobacteria, algae, and plants [119]. PsbP and PsbQ enhance oxygen evolution activity by promoting the binding of Ca^2+^ in the OEC and chloride near the OEC [120]. These subunits are conserved in algae and plants [121]. Mature PSII in cyanobacteria contains PsbO, PsbU, PsbV, and PsbQ [30,122]. The cyanobacterial PsbP homolog is an assembly intermediate [123] and not a component of the mature complex.

PSII reaction centers form supercomplexes with the light-harvesting complex (LHC) antenna proteins (see [3]) (Figure 2). In *Chlamydomonas*, the major complexes mature as trimers and are encoded by a family of nine genes (LHCBM1-9) with high sequence homology [124]. In addition, CP26 and CP29 subunits associate to control the linkage between the PSII core and LHC antenna proteins. Single and double mutants of CP26 and CP29 show impaired photosynthesis and photoprotection [125]. The PSII subunit PsbZ (also known as Ycf9) is also involved in supercomplex assembly, particularly under stress conditions [126].

### 2.6. PSII Phosphorylation and Dephosphorylation

In the chloroplast but not in cyanobacteria, phosphorylation of PSII subunits and the associated antennae plays a role in supercomplex formation and PSII migration within the thylakoid network. These properties affect both protein complex turnover and state transitions [127] (reviewed in [128]). In plants, the STN8 kinase phosphorylates PSII core subunits including D1 [129,130] which can be dephosphorylated by the PBCP phosphatase [131]. In plants, the STN7 kinase phosphorylates LHCs [132] (and to a lesser extent, PSII core subunits [133]) which can be dephosphorylated by the PPH1/TAP38 phosphatase [134,135].

The situation in *Chlamydomonas* is more complicated. The *Chlamydomonas* PSII subunits CP43, D2, and PsbH, but not D1, undergo phosphorylation at their N-termini [136]. The *Chlamydomonas* ortholog of STN8, STL1, has not been fully characterized but is likely to be the PSII core kinase. The phosphorylation of PSII subunits is independent of state transitions, and the specific trigger remains unknown [137]. *Chlamydomonas* LHCs are phosphorylated by the STT7 kinase [138]. Unlike plants which have distinct phosphatases for PSII subunits and LHCs, the *Chlamydomonas* phosphatases CrPPH1 and CrPBCP can dephosphorylate both PSII and LHC [139].

We note that PSII phosphorylation and dephosphorylation mechanisms increase in specificity over evolutionary time. These processes are absent in cyanobacteria. Algae use specialized kinases but redundant phosphatases. Plants use specialized kinases and phosphatases.

### 2.7. Proteolysis of the D1 Subunit

PSII in *Chlamydomonas* is undergoing frequent damage and repair. When PSII is isolated from *Chlamydomonas* cultures grown under optimal conditions, the observed manganese content is lower than expected suggesting that up to 20% of centers are in damaged or assembly states [140,141]. The PSII D1 subunit is most prone to oxidative damage and is rapidly turned over. In *Chlamydomonas* under saturating light conditions, the half-life of D1 is as short as 20 min [142]. While D1 repair is costly in terms of ATP equivalents [143], replacing only this single subunit is more efficient than degrading and reassembling the entire PSII reaction center. D2 turns over at a slightly slower, but significantly rapid rate under high light conditions [144].

The D1 degradation process is well understood in plants where soluble DEG proteases clip loops and the FtsH proteases degrade the resulting fragments. *Arabidopsis* DEG2 clips a stromal D1 loop to generate ~23 kDa and ~10 kDa fragments [145], although D1 is still degraded in the absence of this protease [146]. DEG1 clips a lumenal loop or loops to generate ~16 kDa and ~5 kDa fragments [147]. DEG5 and DEG8 form a complex and DEG8 clips a lumenal loop to generate ~16 kDa and ~18 kDa fragments [148]. In cyanobacteria, DEG proteases are not required for D1 degradation, but do protect cells from heat and light stresses [149].

**Figure 3:**
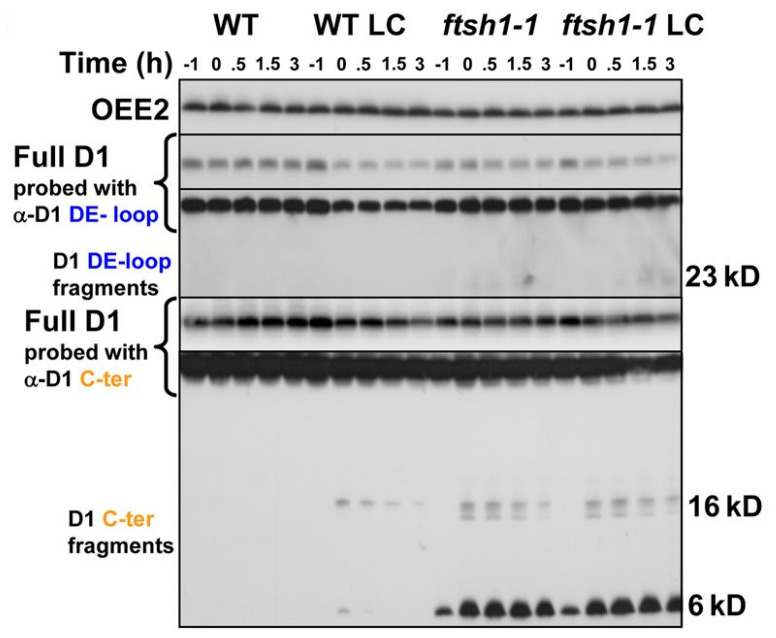
Discrete D1 fragments accumulate in *Chlamydomonas* in an FtsH mutant [150]. Note the appearance of ~23, 16–20 kDa, and 6–10 kDa fragments in the lower panels. (WT: wild type; LC: chloroplast translation inhibitors lincomycin and chloramphenicol added; *ftsh*1–1: mutant with defective FtsH protease due to a FtsH1−R420C mutation; OEE2: loading control probing PsbP). Reproduced with permission from Oxford University Press, Oxford, UK.

FtsH is essential for D1 degradation in *Chlamydomonas*. In a *Chlamydomonas* FtsH mutant strain exposed to light, D1 fragments of ~23 kDa, ~16 kDa, and ~6 kDa accumulate (Figure 3) [150]. However, it is not known which proteases are responsible for these fragments. The *Chlamydomonas* genome encodes 12 DEG proteases with predicted active protease domains and up to seven are predicted to be localized to the chloroplast [151]. DEG1C [152] and DEG9 (unpublished) are active proteases localized to the chloroplast stroma but are not involved in PSII repair or biogenesis. DEG8 and DEG5 are colocalized to the pyrenoid tubules (thylakoids) [51] but have not been further characterized. The identity of the specific protease(s) involved in processing D1 before FtsH remains unknown.

Intriguingly, a 23 kDa D1 fragment is also accumulated in a *Chlamydomonas* double mutant of FtsH and RBD1 in darkness. In this situation, D1 degradation is not the result of photodamage and instead may be induced by a conformational change from the lack of RBD1 [110].

### 2.8. PSII Repair

Following D1 damage, CP43 dissociates from the PSII reaction center, which makes D1 more accessible to proteolysis [81]. A new D1 polypeptide is synthesized and inserted, and CP43 rebinds (reviewed in [153]). This process occurs in non-appressed regions of the thylakoids. In *Chlamydomonas*, the factor TEF30 facilitates D1 insertion and/or CP43 binding during repair of monomeric PSII [154]. Another factor, REP27 (homologous to LPA1 in plants), is also involved in D1 insertion during PSII repair [155,156].

## 3. Conclusions

As shown here, *Chlamydomonas* has remained an important tool for studying PSII from the 1980s to the present. The studies reviewed here have provided deeper insights into biochemical and evolutionary processes:

- PSII assembly in *Chlamydomonas* provides an excellent model system for the evolution and interplay between nuclear and organellar genomes.
- The CES mechanism, which is well studied in terms of PSII assembly in *Chlamydomonas,* is applicable to multiple protein complexes in the chloroplast and other systems.
- Analogously, the extensive translational control of PSII subunits in the *Chlamydomonas* chloroplast has revealed gene regulation strategies.
- The PSII phosphorylation, dephosphorylation, and degradation pathways in *Chlamydomonas* show intermediate mechanisms between cyanobacteria and plants, thus providing insights into evolution of photosynthetic organisms.

## 4. Remaining Questions

While *Chlamydomonas* has clearly contributed much to our understanding of PSII assembly and repair, key questions remain unanswered. These include:

1. What are the molecular mechanisms that allow chloroplast protein import and chloroplast protein synthesis to be coordinated?
2. Why are the genes that encode cytochrome b_559_ separated in the *Chlamydomonas* chloroplast genome?
3. What is the full suite of regulatory elements that control the translation of PSII subunits in the chloroplast?
4. What are the specific triggers for PSII core subunit phosphorylation and dephosphorylation?
5. Which protease(s) degrades D1 into fragments before FtsH processing?

With these questions and others, *Chlamydomonas* will continue to be a practical and powerful system for PSII research.

## Figures and Tables

**Table 1:** Trans-regulatory elements of PSII subunit translation in *Chlamydomonas*.

PSII Subunit Affected	Translation Factor	Mechanism	References
*psbA* (D1)	RB47	Binds to A-rich region in the *psbA* 5′ UTR; required for D1 synthesis	[87,88,89]
	RB60	Protein disulfide isomerase that redox regulates RB47	[87,90]
	TBA1	Oxidoreductase that facilitates binding of RB47 to *psbA* transcript	[91]
	RB55	Observed to bind *psbA* mRNA but not characterized	[92,93]
	RBP63	Binds to *psbA* 5′ UTR; essential for D1 synthesis; subunit of chloroplast pyruvate dehydrogenase complex that becomes a translational regulator upon acetylation	[94,95,96]
	CrHCF173	Homolog of *Arabidopsis* HCF173; affects D1 accumulation	[97]
*psbD* (D2)	NAC1	Promotes *psbD* translation at a step that is likely after initiation	[98,99]
	AC115		
	NAC2	Promotes *psbD* stability by binding to its 5′ UTR	[85,100]
	RBP40 (RB38)	Binds to U-rich region of *psbD* 5′ UTR; forms a complex with NAC2 to control *psbD* mRNA stability and initiation; also observed to bind *psbA* mRNA although this interaction may not be specific	[92,101,102]
*psbB* (CP47)	Mbb1	Promotes *psbB* mRNA stability by interacting with its 5′ UTR; also affects *psbH* mRNA maturation	[103,104]
*psbC* (CP43)	TBC1	Facilitates *psbC* translation by binding to its 5′ UTR	[86,105,106]
	TBC2		
	TBC3		
	MBCI	Stabilizes *psbC* mRNA	[76]

## Data Availability

Not applicable.
